# Modulation of Gut Microbiota by *Lactobacillus casei* Fermented Raspberry Juice In Vitro and In Vivo

**DOI:** 10.3390/foods10123055

**Published:** 2021-12-08

**Authors:** Ting Wu, Xueqi Chu, Yuxin Cheng, Shuxin Tang, Daniel Zogona, Siyi Pan, Xiaoyun Xu

**Affiliations:** Key Laboratory of Environment Correlative Dietology, Ministry of Education, Huazhong Agricultural University, Wuhan 430070, China; ting.wu@mail.hzau.edu.cn (T.W.); chuxueqi2021@163.com (X.C.); yxcheng3@gzu.edu.cn (Y.C.); tangshuxin@webmail.hzau.edu.cn (S.T.); zdanilo27@gmail.com (D.Z.); pansiyi@mail.hzau.edu.cn (S.P.)

**Keywords:** non-dairy probiotic juice, *Lactobacillus* fermentation, polyphenols, antioxidant activity, microbiome

## Abstract

The aim of this study was to investigate the modulation of gut microbiota by fermented raspberry juice (FRJ) both in vitro and in vivo. Results showed that total phenolic content and antioxidant activities of FRJ reached the highest after fermentation for 42 h. Seventeen phenolic compounds were contained in FRJ, mainly including ellagic acid (496.64 ± 2.91 μg/g) and anthocyanins (total concentration: 387.93 μg/g). FRJ modulated the gut microbiota into a healthy in vitro status, with increase of valeric and isovaleric acids production. In healthy mice, all FRJ treatments improved the production of acetic, butyric and isovaleric acids as well as the gene expression of ZO-1, Claudin-1, Claudin-4, Ocdudin, E-cadherin and Muc-2. Moreover, variable gut microbial compositions were found among the groups fed diet-supplemented the different doses of FRJ, within low and median doses of FRJ may regulate the microbiota to a healthier state compared to the high dose supplementation. This study indicated that fermentation is a potential way to produce plant-based juices, which could reshape the gut microbiota and improve the host health.

## 1. Introduction

Raspberry is one of the most popular berries around the world, especially in Europe, America, Australia and China, due to its unique flavour and various health benefits [1]. Raspberries are a rich source of bioactive compounds, such as vitamin A, C, and polyphenols [2]. It was reported that the health benefits of raspberries are primarily mediated by phenolic compounds, mainly ellagitannins, ellagic acid and anthocyanins [3,4]. As a perishable soft fruit, raspberries are often processed and consumed as wines, juices, and jams [5]. However, processing techniques (e.g., thermal processing) may alter the bioactive compounds in raspberries, leading to a decrease in the health-promoting properties of the end products [6]. Thus, it is a challenge for the food industry to find alternative ways to preserve the nutritional and nutraceutical properties of fruit products.

As fruit juices have been revealed to be potential carriers for probiotics [7], fermentation by probiotic lactic acid bacteria (LAB) is a promising way to improve the nutritional characteristics and health-related aspects of plant-based juices [8]. So far, several studies regarding the fermentation of raspberry juice by LAB have been carried out, mainly focused on the changes in chemical compounds, including acid, glucose, and polyphenol contents, as well as in vitro antioxidant capacity [9,10]. However, to our knowledge, no in vivo investigation on the health benefits of LAB-fermented raspberry juice (FRJ) has been published.

Recently, the gut microbiota has attracted intense attention due to its important role in host health [11]. Accumulating evidence supported the effect of berry polyphenols on modulating gut microbiota and decreasing risks of microbiota-associated diseases [12]. Our previous research showed that *Lactobacillus casei* fermentation increased the phenolic contents in blueberry pomace and the fermented pomace improved the fecal microbial community structure [13], which indicates that *L. casei* fermentation may enhance the gut-improving functions of berry products. Whereas, information is lacking regarding the influence of FRJ on gut microbial community structure and functionality. Therefore, the objective of this study is to investigate the gut microbial modulation by *L. casei* FRJ both in vitro and in vivo. As a complementary analysis, antioxidant capacity and effects of FRJ on the gene expressions of colon mechanical barrier were also measured.

## 2. Materials and Methods

### 2.1. Materials

The raspberries (*Rubus idaeus* L.) were kindly provided by Gold berry Co., Ltd. (Tianmen, Hubei, China) and stored at −20 °C for further use. *Lactobacillus casei* (CICC 20280) was used for fermentation.

### 2.2. Preparation and Fermentation of Raspberry Juice

Frozen raspberries were thawed at room temperature, then mixed with distilled water (1:1, *m*/*v*) in a juice blender (300 W) for 1 min. The mixture was homogenized for 5 min, then the whole raspberry juice was collected and stored at 4 °C until further processing.

After pasteurization at 80 °C for 15 min, the sterilized juice was fermented with 5% (*v*/*v*) of *L. casei* in exponential phase at 37 °C for 0, 18, 42 and 72 h without agitation, comprising the fermented raspberry juice (FRJ) group. The non-fermented raspberry juice (NFRJ) group was inoculated with 5% (*v*/*v*) of MRS broth. After fermentation, the bacteria counts and pH values were determined. After centrifuge, FRJ and NFRJ supernatants were collected for antioxidant activity determination.

### 2.3. Determination of Antioxidant Activity

#### 2.3.1. The 2,2′-Azino-bis (3-ethylbenzothiazoline-6-sulfonic acid) Radical Cation Scavenging Activity Assay (ABTS)

The ABTS was carried out following the previous study [14] with slight modification. In brief, the supernatant of FRJ/NFRJ (200 μL) was mixed with 0.8 mL ABTS solution, then incubated in dark at 25 °C for 6 min. The absorbance of the mixture was recorded at 734 nm.

#### 2.3.2. The 2,2-Diphenyl-1-picrylhydrazyl Radical Scavenging Activity (DPPH)

DPPH was measured according to the previous method [15] with some modification. Briefly, supernatant of FRJ/NFRJ (200 μL) was mixed with DPPH solution (1.8 mL, 0.08 mg/mL), incubating in dark at 25 °C for 20 min. Absorbance was recorded at 517 nm.

### 2.4. The Total Phenolic Content (TPC) Analysis

The TPC of FRJ/NFRJ was determined by the Folin–Ciocalteu method. A half milliliter of ciocalteu reagent (50%) was mixed with 1.0 mL of sample. Then Na_2_CO_3_ (5%, 1.0 mL) was added and mixed for 1 min. After incubation (25 °C, 60 min), the absorbance of the mixture was measured at 760 nm.

### 2.5. In Vitro Digestion and Colonic Fermentation

In vitro digestion of 25 mL sample was carried out as described previously [16]. Afterward, the in vitro colonic fermentation of the FRJ/NFRJ digestion(45 mL, equivalent to 10% of diet) was carried out as descried previously [17]. After centrifuging at 7000× *g*, 4 °C for 20 min, precipitates were used for DNA extraction while supernatants were used for short chain fatty acids (SCFAs) analysis, storing at −20 °C until use.

### 2.6. Determination of Phenolic Compounds in FRJ

Phenolic compounds of the FRJ sample were detected using UPLC-MS (Thermo Scientific Q Exactive, Newton Drive, Carlsbad, USA)and analyzed using Xcalibur (Thermo Scientific, Newton Drive, Carlsbad, CA, USA) as described previously [18]. All standards were dissolved to 1 mmol/L by dimethyl sulfoxide respectively, then 50 μL standard solutions were mixed and added the diluent (acetonitrile:water = 3:7, both with 0.1% formic acid-water) to 500 μL.

### 2.7. Animal Study

All the animal procedures and experiments were strictly carried out according to the legislation for care and use of laboratory animals of China and the U.K. Animals (Scientific Procedures) Act (1986). The study was approved by the Animal Ethics Committee of Huazhong Agricultural University (permission no. SYXK (Hubei) 2015-0084). Male Kun Ming mice (17–22 g) were purchased from Huazhong Agricultural University, China, and randomly allocated to 4 groups of 8 animals each. All the mice were provided with a 12 h light/dark cycle under a specific pathogen-free condition with relative humidity (40–60%) and steady temperature (22–24 °C). Diets and sterile water were provided *ad libitum*. For 30 days, each group was fed an experimental diet with different portions of freeze-dried FRJ powders as follows: (1) Standard diet without FRJ supplementation (group C), (2) Standard diet with 3% (wt:wt) FRJ supplementation (group L), (3) Standard diet with 6% (wt:wt) FRJ supplementation (group M), (4) Standard diet with 9% (wt:wt) FRJ supplementation (group H). The doses of freeze-dried FRJ powders, as well as the preparation of experiment diets, were set according to previous studies [19]. Details about the composition of the standard diet AIN93 are shown in Supplementary Information Table S1. The weight and feed utilization rate of the mice were recorded every 2 days.

After 30 days of feeding, the mice were deprived of food for 14 h and then euthanized by diethyl ether inhalation and killed by cervical dislocation. The heart, liver, kidneys and spleen were collected to calculate organ index. All samples were immediately frozen and stored at −80 °C for further analysis.

### 2.8. In Vivo Redox Status Analysis

The hepatic and colonic tissues were homogenized in pre-cooled PBS (*m*/*v* = 1:9) and centrifuged at 10,000× *g*, 4 °C for 10 min. The total antioxidant capacity (T-AOC, A015-1-2) and superoxide dismutase (SOD, A001-1-2) in homogenates were measured using kits for mice (Nanjing Jiancheng Bioengineering Institute, Nanjing, China) following the manufacturer’s instructions.

### 2.9. DNA/RNA Extraction and qPCR

The fecal microbial DNA extraction of precipitates from Section 2.5 and the total RNA extraction of colon tissues from Section 2.7 was performed according to the previous research [18].

The quantity of gut microbiota was amplified by specific primers (Supplementary Information Table S2) of *Akkermansia*, *Bacteroides*, *Bifidobacterium*, butyric acid-producing bacteria, *Escherichia coli*, *Enterococcus*, *Lactobacillus* and *Ruminococcus.* Primers (Supplementary Information Table S3) of ZO-1(zonula occluden-1), Claudin-1, Claudin-4, Occludin, E-cadherin and Muc-2(mucin-2) were designed using BLAST (Basic local alignment search tool) of NCBI (USA) and synthesized by Tsingke Biotech Co., Ltd. (Wuhan, China). The program used for amplification was detailed described in the study of Tang et al. [18]. The relative expression of the intestinal barrier related genes was normalized by internal reference gene GADPH.

### 2.10. Analysis of 16S rRNA Illumina Sequencing

The total bacterial DNA was extracted using Omega Stool DNA Kit (Omega Bio-Tek, USA) according to the manufacturer’s instructions. The V3-V4 regions of bacterial 16S rDNA were amplified by PCR using forward primer (5′-ACTCCTACGGGAGGCAGCAG-3′) and reverse primer (5′-GGACTACHVGGGTWTCTAAT-3′). Samples were sequenced on Illumina HiSeq platforms according to the manufacturer’s manual at Biomarker Technologies Co, Ltd. (Beijing, China).

### 2.11. SCFAs Analysis

Concentrations of 5 SCFAs in supernatants from Section 2.5 and feces from Section 2.7 were determined by gas chromatography according to the previous research [13]. The internal standard (2-ethylbutyric acid) and external standards (acetic, propionic and butyric acids) were purchased from Yuanye Biotech Co., Ltd. (Shanghai, China).

### 2.12. Statistical Analysis

Data were presented as the mean ± standard deviation. ANOVA and Pearson’s correlation analysis were carried out using SPSS 16.0. Statistically significant differences at *p* < 0.05 were calculated using Ducan test.

## 3. Results

### 3.1. Growth of L. casei and pH of FRJ

The number of *L. casei* and the pH value of FRJ with different fermentation time are shown in Supplementary Information Table S4. The density of *L. casei* in FRJ was first decreased from 0 to 18 h, then rebounded to the initial level at 42 h. After 72 h, no colony was observed. The pH value of FRJ was stable around 3.15 during the first 18 h, then significantly dropped to 3.05 at 42 h, and slightly increased to 3.10 at 72 h. For NFRJ, no significant change of the pH was observed during fermentation.

### 3.2. Antioxidant Activities of FRJ and NFRJ

**Figure 1:**
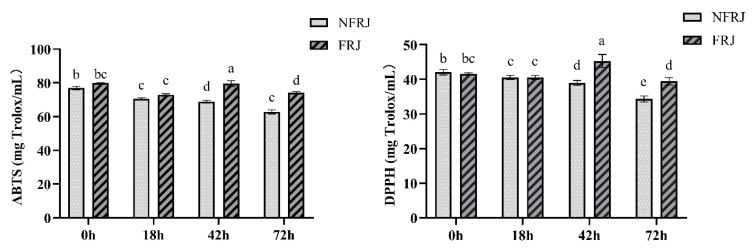
Antioxidant capacities (ABTS and DPPH) of NFRJ and FRJ. Different letters indicated significantly different at *p* < 0.05. ABTS:The 2,2′-azino-bis (3-ethylbenzothiazoline-6-sulfonic acid) radical cation scavenging activity assay; DPPH:The 2,2-diphenyl-1-picrylhydrazyl radical scavenging activity; NFRJ:Non-fermented raspberry juice; FRJ: Fermented raspberry juice.

The antioxidant activities (ABTS and DPPH) of FRJ/NFRJ are listed in Figure 1. Both assays showed the same trend of NFRJ antioxidant activity, which gradually decreased over the time, whereas the antioxidant activity of FRJ reached the highest value at 42 h. In addition, the antioxidant activity of FRJ was significantly higher than that of NFRJ at 42 and 72 h.

### 3.3. The Total Phenolic Content (TPC) of FRJ and NFRJ

**Figure 2:**
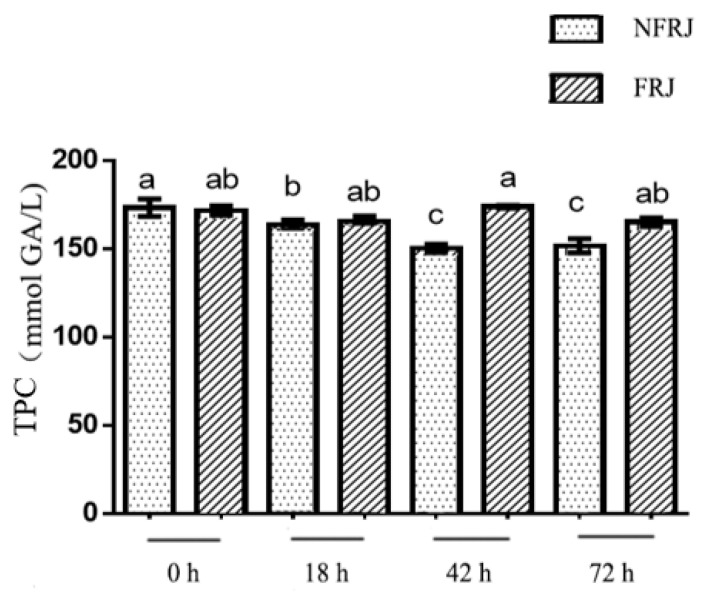
Total phenolic content(TPC) of NFRJ and FRJ. Different letters indicated significantly different at *p* < 0.05.

The TPC of FRJ and NFRJ are displayed in Figure 2. With increasing fermentation time, the TPC of NFRJ decreased while no significant difference in the FRJ TPC was observed. The TPC of FRJ was significantly higher than the TPC of NFRJ at 42 h (173.67 ± 2.16 versus (vs) 150.19 ± 6.04 mmol gallic acid/L) and 72 h (165.00 ± 6.35 vs. 151.64 ± 10.29 mmol gallic acid/L).

### 3.4. Determination of Phenolic Compounds in FRJ

As shown in Table 1, a total of 17 phenolic compounds were quantified in the FRJ, including 5 anthocyanins, 2 flavanols, 4 flavonols, and 6 phenolic acids. The dominant compounds were ellagic acid (496.64 μg/g) and anthocyanins (total concentration: 387.93 μg/g).

### 3.5. Effects of FRJ on Fecal Microbiota In Vitro

After 42 h fermentation and in vitro digestion, the effect of FRJ on 8 fecal bacteria was shown in Table 2. Compared to NFRJ, FRJ increased the abundance of *Escherichia coli*, butyric acid-producing bacteria, *Lactobacillus* and *Akkermansia* in varying degrees, among which the increase of *Lactobacillus* was the highest (5.56 fold). On the other hand, FRJ treatment decreased the abundance of *Bacteroides* and *Ruminococcus.* No significant change in the abundance of *Bifidobacterium* and *Enterococcus* was observed.

### 3.6. Effects of FRJ and NFRJ on In Vitro Production of Short Chain Fatty Acids (SCFAs)

The SCFAs production of in vitro colonic fermentation is presented in Table 3. Compared to NFRJ, treatment with FRJ significantly increased the concentration of valeric (1.63 mmol/L vs. 1.23 mmol/L) and isovaleric acids (8.03 mmol/L vs. 3.94 mmol/L). Whereas, no significant difference in acetic, propionic, and butyric acids production was observed between NFRJ and FRJ treatments.

### 3.7. Effects of FRJ on Growth Performance of Mice

As shown in Supplementary Information Figure S1 and Table S5, there was no significant difference in body weight, feed utilization rate nor organ index among the different groups.

### 3.8. Effects of FRJ on Redox Status in the Liver and Colon of Mice

**Figure 3:**
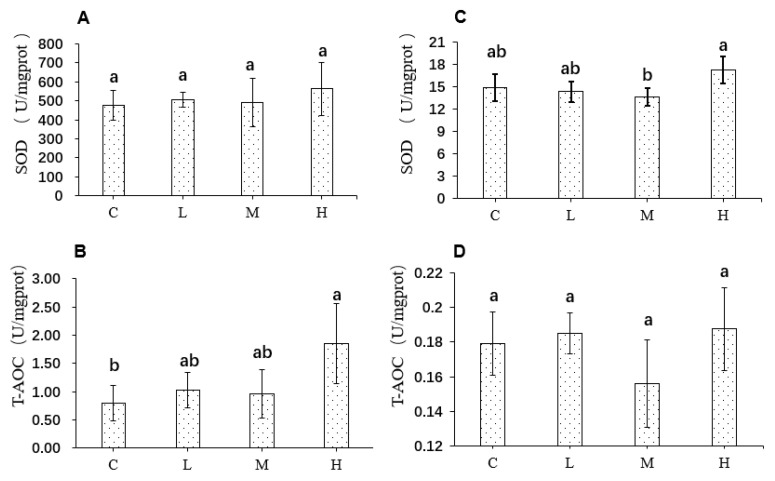
Redox status in the liver (**A**,**B**) and colon (**C**,**D**) of mice after treatments of FRJ. C: mice fed with standard diet; L: mice fed with 3% (wt:wt) FRJ supplementation; M: mice fed with 6% (wt:wt) FRJ supplementation; H: mice fed with 9% (wt:wt) FRJ supplementation. Different letters indicated significantly different at *p* < 0.05. SOD:Superoxide dismutase; T-AOC:Total antioxidant capacity.

The SOD and T-AOC levels in the liver and colon of mice are shown in Figure 3. In the liver, there was no significant difference in SOD level among the four groups, while T-AOC significantly increased in the group treated with high dose FRJ compared to the control group (1.85 ± 0.71 and 0.79 ± 0.31 U/mgprot, respectively). In the colon, no significant difference in T-AOC was observed among the four groups. Also, there was no significant difference in SOD level between the FRJ groups and the control group. However, the SOD of group H was higher than that of group M (17.3 ± 1.8 and 13.7 ± 1.2 U/mgprot, respectively, *p* < 0.05).

### 3.9. Effects of FRJ on Fecal Microbiota In Vivo

**Figure 4:**
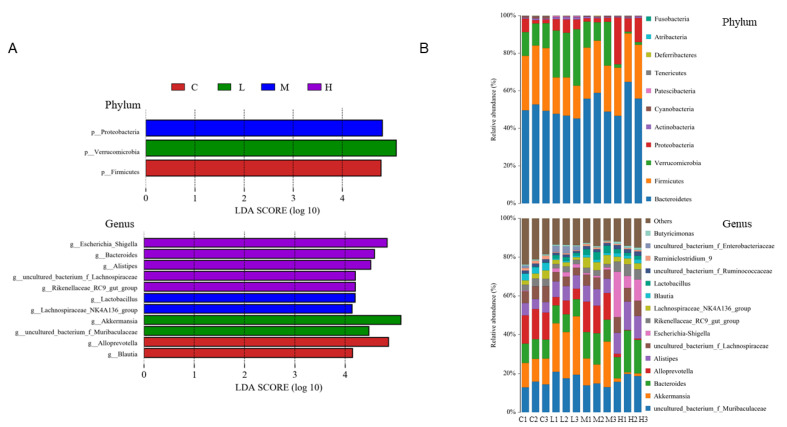
The dominant composition of gut microbiota after FRJ treatments. (**A**) Linear discriminant analysis (LDA) effect size (LEfSe) analyses at phylum and genus level (LDA score > 4.0). (**B**) Relative abundance of microbiota at phylum and genus level. C: mice fed with standard diet; L: mice fed with 3% (wt:wt) FRJ supplementation; M: mice fed with 6% (wt:wt) FRJ supplementation; H: mice fed with 9% (wt:wt) FRJ supplementation. Each number of the samples represents one replicate.

Effects of FRJ on the gut microbiota of mice was evaluated by 16S rRNA Illumina sequence. Although there was no difference in the alpha diversity among all groups (data not shown), the principal component analysis (PCA) showed that FRJ treatments altered the beta diversity of mice, especially in groups L and H, which presented a very distinct cluster from the control group (Supplementary Information Figure S2). Moreover, linear discriminant analysis (LDA) effect size (LEfSe) analysis was performed to identify the statistically significant biomarkers at phylum and genus level of each group (Figure 4A).

The main composition of group M and the control was quite similar, as their dominant phyla were *Bacteroidetes* (50.50% and 54.48%), *Firmicutes* (31.27% and 26.51%), *Verrucomicrobia* (12.55% and 15.68%) and *Proteobacteria* (3.64% and 1.99%), respectively, in descending order. Treatment with FRJ significantly decreased the *Firmicutes* to *Bacteroidetes* ratio in groups L, M, and H (40.99%, 48.76%, 48.80%, respectively) compared to the control group (61.98%). In addition, *Verrucomicrobia* was significantly increased to 26.29% in group L, while considerably dropped to 1.36% in group H. Among all groups, the group H showed the most different gut microbiota with a very high relative abundance of *Proteobacteria* (14.96%).

At the genus level, compared to the control group, all the groups treated with FRJ exhibited a significant decrease in the relative abundance of *Blautia, Ruminiclostridium_9*, and an increase in the relative abundance of *Lactobacillus* (especially group M). *Alistipes, Escherichia-Shigella, Rikenellaceae_RC9_gut_group* and *Butyricimonas* were significantly increased only in group H. On the other hand, a significant increase of *Muribaculaceae* and *Enterobacteriaceae* were only found in group L. Furthermore, the low and high dose FRJ treatments even had some reverse effects. Besides, the abundance of *Akkermansia* markedly increased to 26.29% in the group L (p < 0.05 vs. control) while considerably decreased to 1.36% in the group H (p < 0.05 vs. control).

### 3.10. Effects of FRJ on Production of SCFAs in Mice

As shown in Table 4, four SCFAs were determined and the administration of FRJ increased the production of acetic, butyric and isovaleric acids (*p* < 0.05) while no significant difference in propionic acid production was observed among the four groups. Among the three groups treated with FRJ, only the group M presented a higher content of acetic and butyric acids (378.63 and 395.48 mmol/L, respectively), indicating that the median dose of FRJ improved acetic and butyric acids production in mice.

### 3.11. Effects of FRJ on the Gene Expression of Colon Mechanical Barrier

Table 5 demonstrates that the intake of the 3 different doses of FRJ significantly improved the gene expression of ZO-1, Claudin-1, Claudin-4, Occludin, E-cadherin, and Muc-2 compared to the control. In addition, groups M and H had a better increase of those 6 genes than the group L.

## 4. Discussion

The present study, for the first time, investigated the influence of FRJ on gut microbiota both in vitro and in vivo. It has demonstrated that (i) after *L. casei* fermentation for 42 h, the total phenolic content and antioxidant capacity of raspberry juice was improved in vitro, whereas, only high dose of FRJ intake slightly improved antioxidant activity in vivo, (ii) FRJ reshaped the microbial composition associated with increase in SCFAs production in vitro and in vivo, (iii) the different doses of FRJ had different regulating effects on microbial community structure in vivo. Furthermore, low and median doses of FRJ may regulate the microbiota to a healthier state in the groups L and M compared to the high dose group.

Upon the *L. casei* fermentation, the growth of *L. casei* decreased during the first 18 h. There was also a slight decrease of LAB in *Punica granatum* juice at the early fermentation stage, which was explained by the stress-induced due to the difference of pre-culture and fermentation medium [20]. From 18 h to 42 h, the drop of pH was accompanied by increased microbial growth, probably due to the production of lactic acid by LAB fermentation. It was reported that *L. casei* was able to grow about 2 Log cycles in elderberry juice after 48 h fermentation [21]. However, in the present study, *L. casei* only rebounded to its initial inoculum at 42 h and could no longer survive after 72 h. As it was reported that soluble solids in raspberry juice were the least compared to blackcurrant and red kiwifruit juices [22], raspberry juice may not be a perfect matrix without nutritional supplementation for LAB fermentation.

The antioxidant capacities of the NFRJ determined by ABTS and DPPH decreased over the time in the same trend as the TPC, whereas the *L. casei* fermentation relieved these declines. At 42 h, the antioxidant activity and the TPC of FRJ were both significantly higher than those of NFRJ. Similar results were reported by Ryu et al., showing that TPC, flavonoid content, and DPPH radical scavenging activity of the black raspberry juice increased significantly after LAB fermentation [23]. This positive correlation between antioxidant activity and TPC was also demonstrated in our previous study on blueberry pomace fermented by *L. casei* [13]. The increase of antioxidant capacity may due to the LAB fermentation, as bound polyphenols might be released and degraded into smaller phenolic compounds, exerting higher bioactivity [24]. It is worth noting that, from 42 h to 72 h, the antioxidant activity of FRJ decreased (*p* < 0.05), while TPC was relatively stable. Combined with the fact that no *L. casei* was detected at 72 h, the improvement of antioxidant activity was partly due to *L. casei*. Likewise, previous studies have demonstrated the potential effect of LAB strains on the decrease of oxidative stress and the accumulation of reactive oxygen species [25,26].

The detailed phenolic composition of FRJ was further analyzed by UPLC-MS. Overall, a total of 17 phenolic compounds were detected in the FRJ, among which ellagic acid and anthocyanins were the predominant compounds. Ellagic acid and anthocyanins have been well documented for their antioxidant activity [27]. In this study, they might be the main cause of the increase of FRJ antioxidant activity.

Numerous findings on the gut microbial community suggest the link with the brain, respiratory and urogenital tracts, heart, and skin. Thus understanding the gut microbial structure and function could help to find new approaches to health maintenance [28]. As the TPC and antioxidant capacity of FRJ reached the highest level at 42 h, raspberry juice fermented for 42 h was selected to further investigate its gut microbiota related effects. Firstly, FRJ (equivalent to 10% of diet) was applied to the in vitro digestion and human fecal fermentation. It was found that FRJ had the ability to modulate specific gut microbiota, increasing the abundance of *Lactobacillus* (5.56 fold), *Akkermansia* (1.74 fold), butyric acid-producing bacteria (1.37 fold) and *Escherichia coli* (1.28 fold), while decreasing *Bacteroides* (0.16 fold) and *Ruminococcus* (0.82 fold). As is well known, *Lactobacillus* is an important probiotic, improving gut function, stimulating immune system as well as regulating metabolism [29]. *Akkermansia,* a mucin-degrading bacterium, has been reviewed to have various health benefits, combating diabetes mellitus, obesity, atherosclerosis, cancer, and inflammatory bowel disease [30]. It was reported that *Bacteroides* can weaken gut permeability [31], eventually promoting the migration of enteric pathogens through the intestinal barrier [32]. Consistent with our results, *Lactobacillus* and *Akkermansia* were increased, while *Bacteroidetes* was decreased in cyclophosphamide-induced mice after treatment with litchi juice fermented by *L. casei* [33]. SCFAs are one of the most important microbial metabolites, which have demonstrated positive effects, including inhibition of pathogenic bacteria, maintenance of gut barrier integrity and protection against diet-induced obesity [34,35]. In our in vitro mode, valeric and isovaleric acids were significantly increased by FRJ, whereas acetic, propionic, and butyric acids were not affected. This indicated that FRJ influenced differently the producers of SCFAs. Butyric acid-producing bacteria was promoted by FRJ while *Ruminococcus,* which was also a butyric acid producer [36] was decreased. Overall, FRJ could modulate the gut microbiota community into a healthier status in vitro, with an improvement of SCFAs production to some extent.

We further examined whether FRJ could exert similar effects in vivo, growth performance, redox status in liver and colon, and alterations of the microorganisms in mice fed diet-supplemented with the 3 doses (3%, 6%, and 9% of FRJ). All FRJ treatments had no adverse effects on the growth performance of mice. Moreover, although no significant antioxidant activity of FRJ was found in the colon, all the 3 doses improved the total antioxidant activity of liver tissue with the high dose exerting the strongest effect. These results indicated that FRJ could, to a certain extent, alleviate oxidative stress in vivo. Upon the gut microbial change in vivo, all FRJ treatments reshaped the gut microbiota with different doses gave rise to distinct microbial communities. At phylum level, the relative abundance of *Firmicutes* with respect to *Bacteroidetes* (F/B ratio) was decreased among all the FRJ groups. Since an increment of F/B ratio was related to an imbalance in the taxonomic composition of gut microbiota [37], eventually resulting in metabolic disorders [38], it indicated that the FRJ might promote a healthier state of microbial composition. This hypothesis was supported by facts that all the FRJ doses improved the SCFAs production and the gene expression of ZO-1, Claudin-1, Claudin-4, Ocdudin, E-cadherin, and Muc-2 in mice, which were positively correlated to the intestinal barrier function [19].

At the genus level, the dominant microbiota were *Akkermansia* and *unclassified_Muribaculaceae* for the group L, and *Lactobacillus* and *Lachnospiraceae_NK4A136_group* for the group M, which either are positively correlated with intestinal immune factors and antimicrobial peptides [19] or their implication in the production of acetic and butyric acids [20]. Compared with the low and median doses, high dose of FRJ exerted dramatic different modulation of the initial microbial community. The dominant microbiota in the group H were *Escherichia-Shigella, Bacteroidetes, Alistipes, unclassified_Lachnospiraceae* and *Rikenellaceae_RC9_gut_group,* most of which were reported to be obesity-related bacteria [39]. *Escherichia-Shigella, Bacteroidetes* and *Alistipes* were reported as conditional pathogenic bacteria [40] or potentially harmful bacteria with proinflammatory effects [20]. In addition, the high dose FRJ significantly inhibited *Akkermansia,* whereas the low dose had the opposite effects. In our previous in vitro study on *Lactobacillus plantarum*-fermented mulberry pomace, it was also found that the abundance of *Akkermansia* decreased by high dose of fermented mulberry pomace while increased by low dose treatment [18]. These consistent in vitro and in vivo results indicated that high dose of *Lactobacillus*-fermented berry material inhibits the abundance of *Akkermansia,* by contrast, the low dose promotes the growth. Henning et al. [41] reported that ellagic acid of 10 μM did not inhibit *Akkermansia* while 0.28 mg/mL pomegranate extract (equivalent to 50 μM ellagic acid) significantly inhibited *Akkermansia* growth. As FRJ contains ellagic acids, the observed inhibition effect of the high dose FRJ on *Akkermansia* might due to its high amount of ellagic acid. Since *Akkermansia* can withstand highly oxidative environments [42], another possible explanation for the decrease of *Akkermansia* in the group H could be due to the strongest antioxidant ability observed in the group H compared to the other groups. In summary, treatment with high dose FJR promoted obesity-related bacteria and some potentially harmful bacteria, and inhibited probiotic *Akkermansia* in the group H, whereas treatment with low and median doses FRJ mainly boost some beneficial bacteria in groups L and M. Therefore, low and median doses FRJ could lead to a healthier state of the microbiota. Another study also reported that the gut microbial modulation by 3 different concentrations of cherries juices were significantly different from each other and from the baseline, indicating that dietary modulation of gut microbiota may follow a multi-modal response to nutrient/food doses [43]. Therefore, amounts of juice products or other foods for microbial regulation research need to be well considered and validated.

## 5. Conclusions

The present study demonstrated that FRJ could modulate the profile of gut microbiota both in vitro and in vivo with improvement of antioxidant activity and SCFAs production. Moreover, variable gut microbial compositions were found among the groups fed diet-supplemented the different doses of FRJ, within low and median doses of FRJ may regulate the microbiota to a healthier state compared to the high dose supplementation. Our study indicated that fermentation by *L. casei* is a potential way to produce plant-based juices which could reshape the gut microbiota and improve the host health. However, to realize precise manipulation of gut microbial profile, the amounts of fermented juices need to be considered and validated. Futures studies are needed to understand the underlying mechanisms of the gut microbial change, dynamics of changes in the composition of phenolic compounds in raspberry juice as well as the interplay between gut bacteria and phenolic compounds.

## Figures and Tables

**Table 1:** Phenolic composition in FRJ.

No.	Rt (min)	[M−H]^+^	Error (ppm)	MS^2^	[M−H]^−^	Error (ppm)	MS^2^	Formula	Identification	Concentration (μg/g)
Anthocyanins										
1	5.49	449.10703	−3.020	287				C_21_H_20_O_11_	cyanidin-3-O-glucoside	298.34 ± 2.58
2	5.69	595.16431	−3.335	287				C_27_H_30_O_15_	cyanidin-3-O-rutinoside	10.06 ± 0.05
3	6.30	463.12244	−3.447	301				C_22_H_22_O_11_	peonidin-3-O-glucoside/ peonidin-3-O-galactcoside	2.20 ± 0.01
4	7.38	287.05429	−4.434	287				C_15_H_10_O_6_	cyanidin	58.22 ± 1.44
5	9.31	465.10172	−3.399	303				C_21_H_20_O_12_	delphinidin-3-O-glucoside/ delphinidin-3-O-galactcoside	19.11 ± 0.25
Total Anthocyanins										387.93
Flavanols										
6	6.07				289.07187	2.273		C_15_H_14_O_6_	catechin	0.04 ± 0.00
7	6.47				577.13519	1.021		C_30_H_26_O_12_	procyanidin B	32.17 ± 1.03
Total Flavanols										32.21
Flavonols										
8	5.50				447.09314	0.903	300	C_21_H_20_O_11_	quercetin-3-O-rhamnoside	10.03 ± 0.15
9	9.30				463.08835	1.510	300	C_21_H_20_O_12_	quercetin-3-O-glucoside/ quercetin-3-O-galactcoside	5.07 ± 0.04
10	13.06				301.03534	1.703		C_15_H_10_O_7_	quercetin	16.42 ± 0.20
11	14.97				285.04041	1.744		C_15_H_10_O_6_	kaempferol	0.06 ± 0.00
Total Flavonols										31.58
Phenolic acids										
12	2.55				169.01335	−2.060		C_7_H_6_O_5_	gallic acid	0.12 ± 0.00
13	5.94				137.02328	−4.298		C_7_H_6_O_3_	*p* -hydroxybenzoic acid	0.36 ± 0.02
14	8.53				153.01830	−3.160		C_7_H_6_O_4_	protocatechuic acid	0.27 ± 0.00
15	6.85				179.03415	−1.584		C_9_H_8_O_4_	caffeic acid	0.46 ± 0.01
16	8.81				300.99899	1.821		C_14_H_6_O_8_	ellagic acid	496.64 ± 2.91
17	13.38				147.04410	−3.430		C_9_H_8_O_2_	cinnamic acid	0.29 ± 0.05
Total phenolic acids										498.14

Rt, retention time; [M−H]^+^, precursor ion obtained from positive mode; [M−H]^−^, precursor ion obtained from negative mode; MS^2^, fragment ions of precursor ion obtained from tandem mass spectrum.

**Table 2:** The relative abundance of fecal microbiota with FRJ and NFRJ treatment.

	*Bacteroides*	*Bifidobacterium*	*Ruminococcus*	*Escherichia coli*	Butyrate- Producing Bacteria	*Lactobacillus*	*Enterococcus*	*Akkermansia*
NFRJ	1.00 ± 0.00 ^a^	1.00 ± 0.00 ^b^	1.00 ± 0.00 ^a^	1.00 ± 0.00 ^c^	1.00 ± 0.00 ^b^	1.00 ± 0.00 ^c^	1.00 ± 0.00 ^a^	1.00 ± 0.00 ^b^
FRJ	0.16 ± 0.02 ^b^	1.14 ± 0.12 ^b^	0.82 ± 0.14 ^b^	1.28 ± 0.42 ^b^	1.37 ± 0.22 ^a^	5.56 ± 0.19 ^b^	1.06 ± 0.14 ^a^	1.74 ± 0.14 ^a^

Different letters indicated significantly different at *p* < 0.05. The NFRJ sample was used as the control, of which the relative abundance of the microbiota was set as 1.00.

**Table 3:** SCFAs production of in vitro colonic fermentation with FRJ and NFRJ treatments.

Group	Acetic Acid (mmol/L)	Propionic Acid (mmol/L)	Butyric Acid (mmol/L)	Valeric Acid (mmol/L)	Isovaleric Acid (mmol/L)
NFRJ	275.07 ± 24.38 ^a^	5.37 ± 1.86 ^a^	16.69 ± 2.06 ^a^	1.23 ± 0.23 ^b^	3.94 ± 0.24 ^b^
FRJ	266.83 ± 12.92 ^a^	4.54 ± 0.23 ^ab^	17.75 ± 0.62 ^a^	1.63 ± 0.05 ^a^	8.03 ± 0.29 ^a^

Different letters indicated significantly different at *p* < 0.05.

**Table 4:** SCFAs production of mice after treatments of FRJ.

Group	Acetic Acid (mmol/L)	Propionic Acid (mmol/L)	Butyric Acid (mmol/L)	Isovaleric Acid (mmol/L)
C	281.42 ± 8.29 ^c^	331.57 ± 60.75 ^a^	308.10 ± 42.75 ^b^	162.46 ± 12.59 ^b^
L	358.65 ± 14.44 ^b^	366.74 ± 57.29 ^a^	350.27 ± 17.38 ^ab^	169.08 ± 31.04 ^b^
M	378.63 ± 40.78 ^a^	377.61 ± 19.52 ^a^	395.48 ± 20.66 ^a^	207.00 ± 11.03 ^a^
H	342.17± 8.40 ^b^	362.43 ± 30.35 ^a^	374.40 ± 23.03 ^a^	202.75 ± 11.96 ^a^

C: mice fed with standard diet; L: mice fed with 3% (wt:wt) FRJ supplementation; M: mice fed with 6% (wt: wt) FRJ supplementation; H: mice fed with 9% (wt:wt) FRJ supplementation. Different letters indicated significantly different at *p* < 0.05.

**Table 5:** Effects of FRJ on gene expression of mechanical barrier of colon.

Group	ZO-1	Claudin-1	Claudin-4	Occludin	E-cadherin	Muc-2
C	1.00 ± 0.00 ^a^	1.00 ± 0.00 ^a^	1.00 ± 0.00 ^a^	1.00 ± 0.00 ^a^	1.00 ± 0.00 ^a^	1.00 ± 0.00 ^a^
L	1.41 ± 0.13 ^b^	1.31 ± 0.13 ^b^	1.30 ± 0.06 ^b^	1.13 ± 0.08 ^b^	1.19 ± 0.09 ^b^	1.49 ± 0.21 ^b^
M	1.82 ± 0.06 ^c^	1.48 ± 0.13 ^c^	1.43 ± 0.08 ^c^	1.33 ± 0.11 ^c^	1.35 ± 0.24 ^b^	1.82 ± 0.33 ^c^
H	1.58 ± 0.10 ^bc^	1.61 ± 0.04 ^d^	1.42 ± 0.12 ^c^	1.32 ± 0.05 ^c^	1.30 ± 0.07 ^b^	1.67 ± 0.18 ^bc^

C: mice fed with standard diet; L: mice fed with 3% (wt:wt) FRJ supplementation; M: mice fed with 6% (wt:wt) FRJ supplementation; H: mice fed with 9% (wt:wt) FRJ supplementation. Different letters indicated significantly different at *p* < 0.05.

## Supplementary Materials

The following are available online at https://www.mdpi.com/article/10.3390/foods10123055/s1, Figure S1: Body weight change (left) and feed utilization rate (right) of mice in each group, Figure S2: PCA analysis of fecal microbiota after FRJ treatments with different doses., Table S1: Compositions of the standard diet AIN93, Table S2: Primers of gut microbiota used in this study, Table S3: Primers of related genes used in this study, Table S4: Changes of total number of L.casei colonies and pH of NFRJ/FRJ, Table S5: Organ index of mice in each group
