# Supplementary material for: Modulation of Gut Microbiota by Lactobacillus casei Fermented Raspberry Juice In Vitro and In Vivo
Source: Foods. 2021 Dec 8;10(12):3055. doi: 10.3390/foods10123055 (PMC8702086; doi:10.3390/foods10123055)
Supplement: Supplementary file 1 [file foods-10-03055-s001.zip › foods-1467680-supplementary.pdf]

Supplementary Information

Table S1. Compositions of the standard diet AIN93.

Composition	Feed
Energy	3766 Kcal/kg
Water	6.6%
Fat	7%
Carbohydrate	64.3%
Protein	17.8%
Ash	4.17%

Table S2. Primers of gut microbiota used in this study.

Name	Primers
Bacteroides	F:ATAGCCTTTCGAAAGRAAGAT<br>R:CCAGTATCAACTGCAATTTTA
Escherichia coli	F:GTTAATACCTTTGCTCATTA<br>R:ACCAGGGTATCTTAATCCTGTT
Enterococcus	F:CCCTTATTGTTAGTTGCCATCAT<br>R: ACTCGTTGTACTTCCCATTGT
Bifidobacterium	F: GGGTGGTAATGCCGGATG<br>R: TAAGCGATGGACTTTCACACC
Ruminococcus	F:TTAACACAATAAGTWATCCACCTGG<br>R:ACCTTCCTCCGTTTTGTCAAC
Lactobacillus	F: AGCAGTAGGGAATCTTCCA<br>R: CACCGCTACACATGGAG
Akkermansia	F:CAGCACGTGAAGGTGGGGAC<br>R:CCTTGCGGTTGGCTTCAGAT
Butyrate-producing	F:GCIGAICATTTCACITGGAAYWSITGGCAYATG

bacteria	R:CCTGCCTTTGCAATRTCIACRAANGC
Internal reference	F:ACTCCTACGGGAGGCAGCAG<br>R:GGACTACHVGGGTWTCTAAT

Table S3. Primers of related genes used in this study.

Name	Primers
GADPH	F:GGAGCGAGATCCCTCCAAAAT<br>R:GGCTGTTGTCATACTTCTCATGG
ZO-1	F:CGGAACTATGACCATCGCCTAC<br>R:CTTCGGGATGTTGTCTGGAGTC
Claudin-1	F:GGGCTGATCGCAATCTGGAGTC<br>R:CCACTAATGTCGCCAGACCTGA
Claudin-4	F:GGCTGAGCGATGGCGTCTAT<br>R:CGATGTTGCTGCCGATGAAGG
Occludin	F:TTCCACACTTGCTTGGGACAGA<br>R:TCCGCCATAGCCATAGCCATAG
E-cadherin	F:GCCATCGCCTACACCATCGT<br>R:GCAGCCTGAACCACCAGAGT
Muc-2	F:ACGCCTGTGACCTCTCAATCC<br>R:CCGCTGATGAAGTGACGAATGG

Table S4. Changes of total number of *L.casei* colonies and pH of NFRJ/FRJ

Time (h)	NFRJ (lg CFU/mL)	FRJ (lg CFU/mL)	NFRJ-pH	FRJ-pH
0	ND	7.37 ± 0.05^{a}	3.17 ± 0.01^{a}	3.14 ± 0.04^{a}
18	ND	6.15 ± 0.21^{b}	3.26 ± 0.05^{a}	3.15 ± 0.02^{a}
42	ND	7.12 ± 0.13^{a}	3.13 ± 0.01^{a}	3.05 ± 0.01^{b}
72	ND	ND	3.17 ± 0.07^{a}	3.10 ± 0.05^{ab}

Data expressed as mean ± standard deviation; Data with different letters in each column were significantly different at $p < 0.05$; ND: not detected.

Table S5. Organ index of mice in each group

Group	Heart (g/kg)	Liver（g/kg)	Spleen（g/kg)	Kidney（g/kg)
C	5.79 ±0.60[a]	37.41 ±2.73[a]	2.01 ±0.33[a]	12.45 ±1.30[a]
L	6.41 ±0.47[a]	41.12 ±3.10[a]	1.85 ±0.30[a]	12.40 ±1.03[a]
M	6.58 ±0.53[a]	39.04 ±2.67[a]	1.79 ±0.53[a]	12.80 ±1.39[a]
H	6.65 ±1.50[a]	41.35 ±0.81[a]	1.90 ±0.30[a]	12.86 ±0.18[a]

Group C: mice fed with standard diet; Group L: mice fed with 3% (wt:wt) FRJ supplementation; Group M: mice fed with 6% (wt:wt) FRJ supplementation; Group H: mice fed with 9% (wt:wt) FRJ supplementation. Data expressed as mean ± standard deviation; Data with different letters in each column were significantly different at $p < 0.05$.

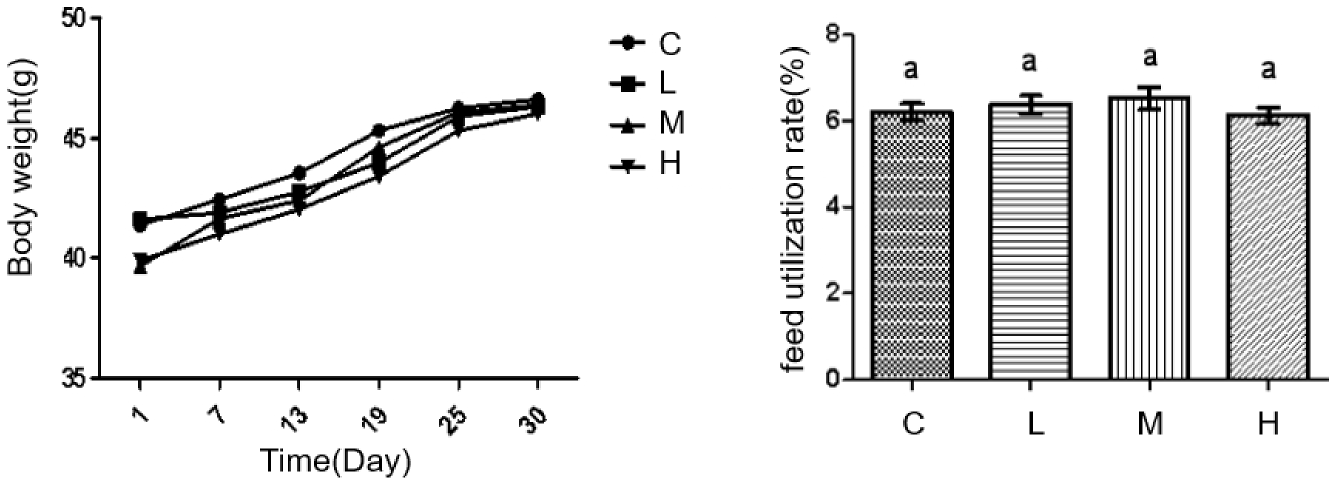

Figure S1. Body weight change (left) and feed utilization rate (right) of mice in each group. Group C: mice fed with standard diet; Group L: mice fed with 3% (wt:wt) FRJ supplementation; Group M: mice fed with 6% (wt:wt) FRJ supplementation; Group H: mice fed with 9% (wt:wt) FRJ supplementation.

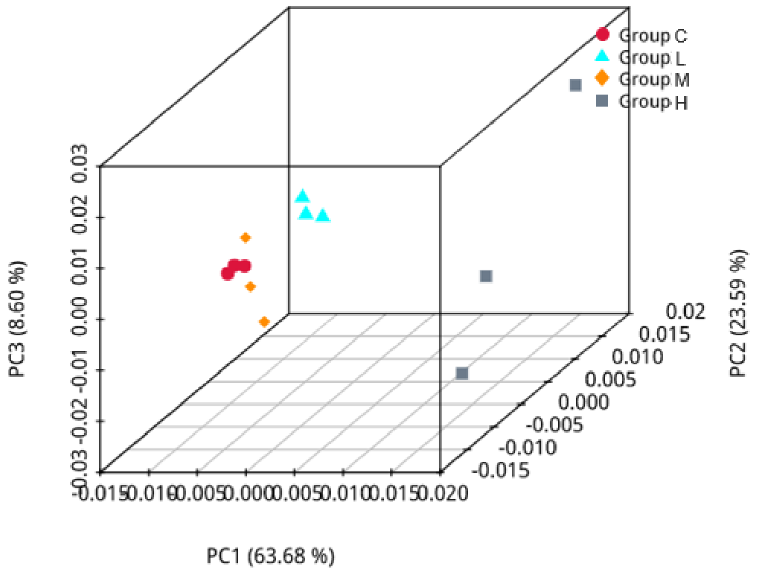

Figure S2. PCA analysis of fecal microbiota after FRJ treatments with different doses. The PCA reflected the differences and distances of samples, by analyzing the OTU (97% similarity). The closer the two samples are, the more similar the composition of the two samples is. Group C: mice fed with standard diet; Group L: mice fed with 3% (wt:wt) FRJ supplementation; Group M: mice fed with 6% (wt:wt) FRJ supplementation; Group H: mice fed with 9% (wt:wt) FRJ supplementation.
